# Food Fortification Using Spray-Dried Emulsions of Fish Oil Produced with Maltodextrin, Plant and Whey Proteins—Effect on Sensory Perception, Volatiles and Storage Stability

**DOI:** 10.3390/molecules27113553

**Published:** 2022-05-31

**Authors:** Annelie Damerau, Sari A. Mustonen, Dorota Ogrodowska, Laura Varjotie, Waldemar Brandt, Oskar Laaksonen, Małgorzata Tańska, Kaisa M. Linderborg

**Affiliations:** 1 Food Sciences, Department of Life Technologies, University of Turku, 20014 Turku, Finland; annelie.damerau@utu.fi (A.D.); saanmus@utu.fi (S.A.M.); laura.e.varjotie@utu.fi (L.V.); osanla@utu.fi (O.L.); 2 Department of Food Plant Chemistry and Processing, Faculty of Food Science, University of Warmia and Mazury, 10-718 Olsztyn, Poland; dorota.ogrodowska@uwm.edu.pl; 3 Department of Dairy Science and Quality Management, Faculty of Food Science, University of Warmia and Mazury, 10-719 Olsztyn, Poland; waldemar.brandt@uwm.edu.pl

**Keywords:** microencapsulation, omega-3 fatty acids, functional foods, shortbread cookies, dark chocolate, HS-SPME-GC-MS

## Abstract

Fortification of foods with fish oil rich in *n*–3 fatty acids improves the nutritional value, but creates challenges with flavor and oxidative stability, especially during storage. Pea, soy, and sunflower proteins were used in combination with whey protein or maltodextrin to encapsulate fish oil by spray-drying. The use of whey protein compared with maltodextrin as wall material improved oxidative stability of spray-dried emulsions, although the use of whey protein increased the number of observed cracks in outer shell of the particles. Non- and encapsulated oil were used in cookies and chocolates to examine flavor characteristics by generic descriptive analysis and volatile products by solid-phase microextraction with gas chromatography-mass spectrometry. A long-term storage test at room temperature was conducted to evaluate the oxidative stability of the food models. Fortification changed the texture, odor, and flavor of the food models with fishy flavor being the most impactful attribute. For both food models, use of pea protein with maltodextrin resembled attributes of control the best. Fortification and encapsulation material also affected volatile profiles of food models. Both non-encapsulated oil and whey protein formulations performed well in regard to oxidative stability for both food models. Generally, the cookie model showed more potential for fortification than the chocolate one.

## 1. Introduction

Fish oil in the form of either fatty fish or oil supplement is the main source of *n*–3 long chain polyunsaturated fatty acids (PUFAs), most importantly eicosapentaenoic acid (20:5*n–*3, EPA) and docosahexaenoic acid (22:6*n–*3, DHA), in the human diet [1]. These *n–*3 PUFAs play an important role in the cardiovascular system and inflammatory balance and are essential for normal brain and eye development [2]. Although the health benefits of *n*–3 PUFAs are well established, their dietary intake is not at the recommended level [3,4].

The low intake of long chain PUFAs relates to the fishy smell and flavor of most supplements [5,6]. *n*–3-enriched foods are an alternative to fish and fish oil supplements. In Europe, foods with 0.3 g alpha-linolenic acid per 100 g and per 100 kcal, or at least 40 mg of the sum of EPA or DHA per 100 g and per 100 kcal can be claimed as a source of *n–*3 fatty acids [7]. According to the same regulation, a claim that a food is high in *n–*3 fatty acids can be made when the product contains at least 0.6 g alpha-linolenic acid per 100 g and per 100 kcal, or at least 80 mg of the sum of EPA or DHA per 100 g and per 100 kcal. However, fortification with fish oil is challenging as EPA and DHA are prone to lipid oxidation. Lipid oxidation decreases the nutritional and sensory quality, and can even result in the formation of potentially toxic compounds [8,9]. Further, the natural flavor and polarity of fish oil may place limits on the potential product types that can be fortified [10].

Microencapsulation of oil could address these challenges [10]. The most common microencapsulation technique used for fish oil is spray drying. In the resulting powder, the encapsulated core is protected from oxygen and pro-oxidants by a wall material commonly consisting of carbohydrates and/or proteins [11,12], and thus improved oxidative stability is achieved [13]. Microencapsulation can mask fishy flavor [14], and dry powder is more convenient in industrial processes than liquid oil [15], allowing a wider selection of foods for fortification.

The challenges and opportunities for *n*–3 food fortification have been previously reviewed, in general by Ganesh and Hettiarachchy [10] and focusing on fortification by fish oil by Jamshidi et al. [16]. Although previous studies exist on microencapsulation of fish oils, incorporation of the capsules into foods has been scarcely studied. Biopolymers used as wall materials may dissolve in liquid and semi-liquid foods, turning them into fish oil emulsion, which no longer provides the oil protection from pro-oxidants. Thus, the main target foods for encapsulated fish oil are solid foods like bars, infant powder formulas, and baked products [12]. For example, Jeyakumari et al. [17] incorporated fish oil or fish oil microencapsulates in cookies. While microencapsulation improved the oxidative stability of fish oil in the cookies determined by the thiobarbituric acid (TBA) value, sensory evaluations showed a negative impact on the texture and taste of several formulations. However, plant proteins were not used in this study as part of wall material formulation. Finding the encapsulation formulation and food matrix combination that provides both sensory quality as well as oxidative stability is likely to be the main challenge in fortification. 

The hypothesis of this study was that by using plant proteins (pea, soy, and sunflower proteins) in combination with other proteins or carbohydrates in encapsulation by spray drying, the fish oil flavor could be masked and oxidative stability of fish oil improved in fortified foods. Therefore, the main objectives were to develop fortified food models (shortbread cookies and dark chocolate) containing encapsulated fish oil using different protein/carbohydrate or protein/protein combinations as wall material and to investigate the sensory perception and volatile profiles of the developed food concepts. Shortbread cookies and dark chocolate were chosen as food models based on their low water activity and long shelf-life. Prior to addition to the food models, the surface and total oil content of the spray-dried emulsions (SDEMs) were determined gravimetrically and fatty acid composition was analyzed using gas chromatography (GC) with a flame ionization detector (FID). Further, oxidative stability index (OSI) and volatile secondary oxidation products (VSOPs) using headspace solid-phase microextraction (HS-SPME) coupled GC with mass spectrometer (MS) were analyzed to characterize oxidative stability of SDEMs. Only the most promising formulations were used in both food models in different concentrations, analyzed by HS-SPME-GC-MS and evaluated by a trained sensory panel. Finally, a long-term storage test at room temperature was conducted to evaluate the oxidative stability based on VSOPs of the food models. 

## 2. Results and Discussion

### 2.1. Characterization of SDEMs

All plant protein containing SDEMs with whey protein concentrate (W) had a higher total oil content than corresponding emulsions containing maltodextrin (M). The total oil content, ranging from 38.62% to 44.18%, was lower than predicted based on the composition for the emulsions, suggesting small losses of oil during processing and incomplete water evaporation during spray-drying, especially in samples containing M (Table 1). M can absorb more water than W because it forms an outer layer on the droplet, which alters the surface stickiness of particles due to the transition into a glassy state [18]. W, combined with sunflower protein (SF) and pea protein (P), had lower content of surface oil (SFW 13.39% and PW 15.90%) compared with samples with M (SFM 15.87% and PM 17.35%). In the soy protein (S)-containing sample the surface oil content was comparable. Wang et al. [19] reported that the whey protein isolate content in the emulsion increased the oil retention principally by reducing the time to form a semi-permeable crust at the droplet–air interface during spray-drying, making it difficult for the oil to diffuse to the particle surface during drying. Encapsulation efficiency (EE) values were relatively high for powders with such high oil content (57.27% (PM) to 69.64% (SFW)). High oil-loading microcapsules (1:1) have generally low EE, affecting the higher surface oil content of the particles [19,20]. The findings are in agreement with those of Di Giorgio et al. [21], where EE ranged from 57.73% (1:1 = protein:oil mass ratio) to 88.74% (4:1 = protein:oil mass ratio).

The fatty acid composition of SDEMs was comparable with that of non-encapsulated fish oil (FO) (Table 2). The DHA content ranged from 14.29% to 14.57% while the EPA content ranged from 9.77% to 10.22% in SDEMs, respectively. The content in the native oil was slightly higher with 14.69% and 10.34% of DHA and EPA, respectively. However, these differences were not significant. Castejón et al. [22] confirmed that encapsulation by spray drying did not affect omega-3 fatty acid profile of lipids extracted from oilseeds and microalgae.

**Figure 1:**
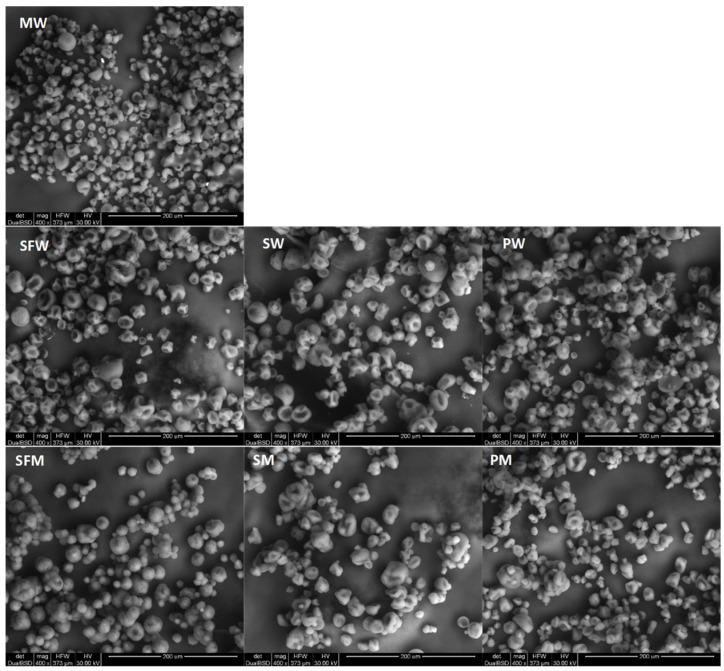
Scanning electron microscopy images for spray-dried emulsions formulated with M = maltodextrin, W = whey protein concentrate, SF = sunflower protein, S = soy protein, or P = pea protein as wall material.

Generally, scanning electron microscopy (SEM) images showed typical well-separated particles similar to irregular-shaped beads (Figure 1). However, agglomerates were visible in the PW sample. The samples were not uniform in size, which can be explained by the high oil:coating material ratio. Rodea-González et al. [23] reported that as the core to wall material ratio decreased, more uniformly sized microcapsules were formed. Pea protein containing powders consisted of a large number of small particles (Figure 1) with the greatest resemblance to the MW sample. Gharsallaoui et al. [24] presented SEM images of powders of oil obtained by using 0.25% pea protein isolate and 11% starch hydrolysates with various dextrose equivalents (DE) as wall materials. In their study, microcapsules obtained from DE-6 were dented and partially disrupted, while powders obtained from DE-19 and DE-28 were slightly circular and uniform, showing minimum cracks and dents on the surface. The particles of the powders containing M were smoother and more spherical than those containing W. The highest number of cracks was found in the SFW, SW, and PW samples.

All SDEMs were more stable than FO (OSI 1.36 h) (Table 1) of which PW was the most stable (OSI 15.7 h). Although amino acids with the highest antioxidant capacity, i.e., the sulfur-containing amino acids (cysteine, methionine, and tryptophan) are not abundant in pea proteins, they contain comparably high amounts of hydrophobic and aromatic amino acids, such as proline, valine, leucine, isoleucine, and phenylalanine, which also possess antioxidant activity. Generally, the majority of antioxidative amino acids are inaccessible in unmodified pea protein due to association and molecular tertiary structure. However, enzymatic hydrolysis may expose these amino acids [25]. There was no correlation between OSI and surface lipid content. Volatile profiles of SDEMs were analyzed, and VSOPs were selected based on abundance and origin. Selected VSOPs were propanal, 2-ethylfuran, hexanal, 2-hexenal (*E)*, 2-pentylfuran, 3,5-octadien-2-one (*E,Z/E,E*), and nonanal, which are known oxidation products of fish oil [13,26]. The total peak area of selected VSOPs was in line with OSI (Table 1) with PW having the lowest formation of selected VSOPs and the highest OSI, and SFM having the highest formation of selected VSOPs and the lowest OSI. It can be concluded that the use of W compared with M as wall material improved oxidative stability of SDEMs, although the use of W increased the number of observed cracks in SDEMs. SDEMs with OSI over 10 h and a total peak area of selected VSOPs under 30 × 10^5^ counts per s (Table 1) were regarded as stable enough for food processing. Although, failing the quality parameters set, SM was included in order to study the effect of the use of M vs W in wall material composition during fortification. Therefore, for the fortification, MW, SW, PW, SM, and PM and FO were used. Further, a control (C) without any addition of fish oil was studied to determine the matrix effects of the food models.

### 2.2. Sensory Analysis

#### 2.2.1. Performance of the Assessors and the Panel

The panel agreed on fishy flavor in both food models. A high agreement was also attained on crumbliness and melting texture in chocolates, and on fishy odor and flavor intensity in cookies. The odor intensity and bitter taste were the most difficult for the panel. The panel also had some disagreement on sweetness and oily taste in shortbread cookies and on aftertaste in chocolates. Some assessors had difficulties in differentiating chocolates based on fishy odor, while for others the difference was clear. However, none of the participants performed systematically poorly, due to which the results of all assessors were included in subsequent statistical analyses.

#### 2.2.2. The Effect of SDEMs on Perceived Sensory Differences

The most notable differences between samples appeared in fishy odor and flavor in both chocolates and cookies (Appendix A; Table A1 and Table A2). Additionally, differences were found in melting sensation and crumbliness in chocolate (Appendix A; Table A1), and in odor and flavor intensity, softness, and moistness in cookies (Appendix A; Table A2). There were no differences between SW and SM cookies in fish flavor in lower (1.5%) or higher (3.0%) oil content, whereas PW was estimated to have a fishier flavor than PM at both oil contents. In the study by Hughes et al. [27], increased fish oil content decreased the aroma, flavor, and texture acceptability scores of fortified nutrition bars. Additionally, whole wheat bread enriched with higher content of microencapsulated *n*–3 fatty acids correlated with more intense fishy flavor and lower acceptability scores [28]. Increasing the amount of encapsulated oil in cookies from 1.5% to 3.0% increased the fishy flavor for SM, SW, and PW, however not for PM. Differences between soy and pea proteins became more prominent in the higher oil content. Cookies containing SM were generally evaluated as fishier than PM. However, PW produced a fishier flavor than SW. In chocolate, the use of S and W increased the perceived fishy flavor. The fishy flavor-enhancing effect of W may result from higher total oil levels than M-containing SDEMs. Additionally, smoother powders containing M are potentially better at hiding the fishiness than cracked ones containing W.

In chocolate, SW had a stronger fishy odor than PW. The same was also observed in cookies with SM being fishier than PM. Soy protein itself may have an off-flavor [29], which may raise fishy odor when interacting with matrix. Overall, the fishy-enhancing effect of S in cookies was most pronounced at the higher oil content. Additionally, compared with M, W increased fishy odor when combined with P (in shortbread cookies) and S (in chocolate).

The addition of fish oil in all forms reduced the sensation of chocolate melting and increased crumbliness. However, M added less crumbliness than W. In cookies, M decreased the perceived softness compared with C and the cookies containing FO. Additionally, González et al. [30] reported bread enriched with microencapsulated chia oil was harder than bread with non-encapsulated oil. However, there was no statistically significant difference between control and microencapsulated bread. This indicates that in addition to wall materials, the food matrix plays a key role in the effect of microcapsules on the texture.

#### 2.2.3. Sensory Profiles

**Figure 2:**
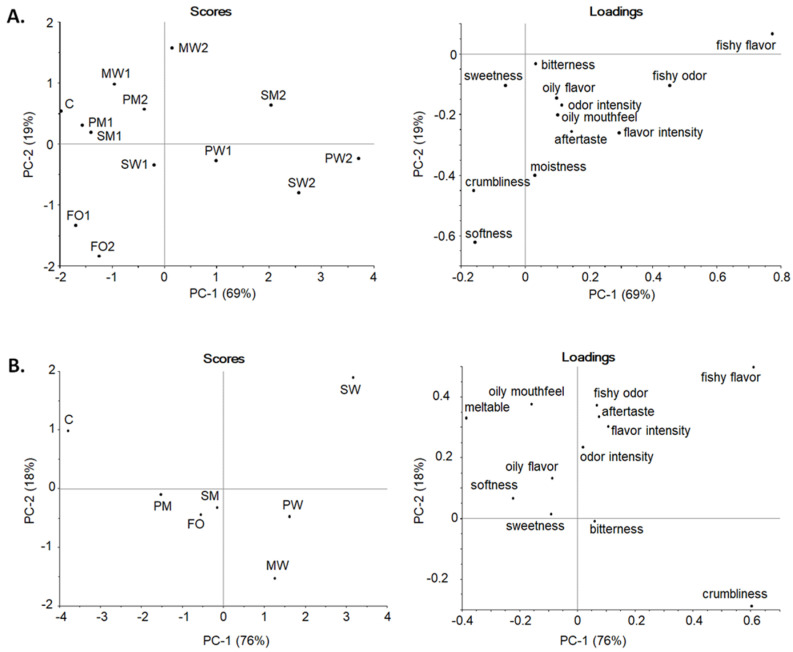
Consensus PCA scores and loadings plots of the descriptive sensory profile of (**A**). shortbread cookies and (**B**). chocolate. In scores plot: C = control, FO = fortified with non-encapsulated fish oil, M = maltodextrin, W = WPC = whey protein concentrate, S = soy protein, or P = pea protein as wall material, 1 = 1.5% oil, and 2 = 3.0% oil.

In the consensus principal component analysis (PCA) (Figure 2A), PC-1 explained 69% and PC-2 19% of the variance among cookies. The fishy flavor and fishy odor had a positive loading on PC-1. Softness, crumbliness, and moisture had negative loadings on PC-2 in which the cookies were evenly distributed. FOs were the softest and MWs were the hardest. SDEM cookies with a higher oil content had a positive load on PC-1, indicating them to be fishier, while cookies with less oil had not as clear fishiness. However, pea protein cookies made an exception: PW1 and PW2 had distinct fishiness while PM1 and PM2 showed minor fishiness. The PCA pea protein and maltodextrin combination resembled the most C and was the best choice for cookies enriched with SDEMs. FO cookies were not perceived as fishy, even though the oil was not protected by encapsulation, as was also noticed in the study of Hughes et al. [27] in which a small amount of fish oil in the product remained undetected and did not affect the acceptability.

For the chocolates, PC-1 explained 76% and PC-2 18% (Figure 2B). Fishy flavor and crumbliness had positive loadings on PC-1, while fishy odor, aftertaste, and oily mouthfeel had an average loading on PC-2. W addition on chocolate increased crumbliness and fishy flavor. Especially SW had strong fishiness as was also noted in general linear model analysis of variance (ANOVA) calculations (Appendix A; Table A2). Although maltodextrin/plant protein combinations and FO chocolates formed a separate group from W samples, all enriched chocolates differed from C. However, maltodextrin seems to be the most promising alternative to for enriched chocolates, especially combined with pea protein.

### 2.3. Volatile Composition of Food Models

From the fresh cookie model, 37 volatile compounds were identified (Table 3), of which 2-heptanone was the most abundant followed by 2-nonanone, 2,4-dimethylheptane, and acetic acid. The only volatiles not also found in C were propanal and maltol. Propanol is a typical volatile oxidation product formed from *n*–3 PUFAs and maltol is a product of the early stages of the Maillard reaction. Additionally, other Maillard reaction products were detected in the cookie model due to the baking process such as furfural, 2-furanmethanol, and 3-dihydro-3,5-dihydroxy-6-methyl-4*H*-pyran-4-one. Similar compounds have been detected in corresponding models previously [31,32]. Typical volatiles originating from butter were, e.g., 2,3-butanedione, butanoic acid, hexanoic acid, nonanal, and δ-hexalactone, as also found previously [33,34,35].

**Figure 3:**
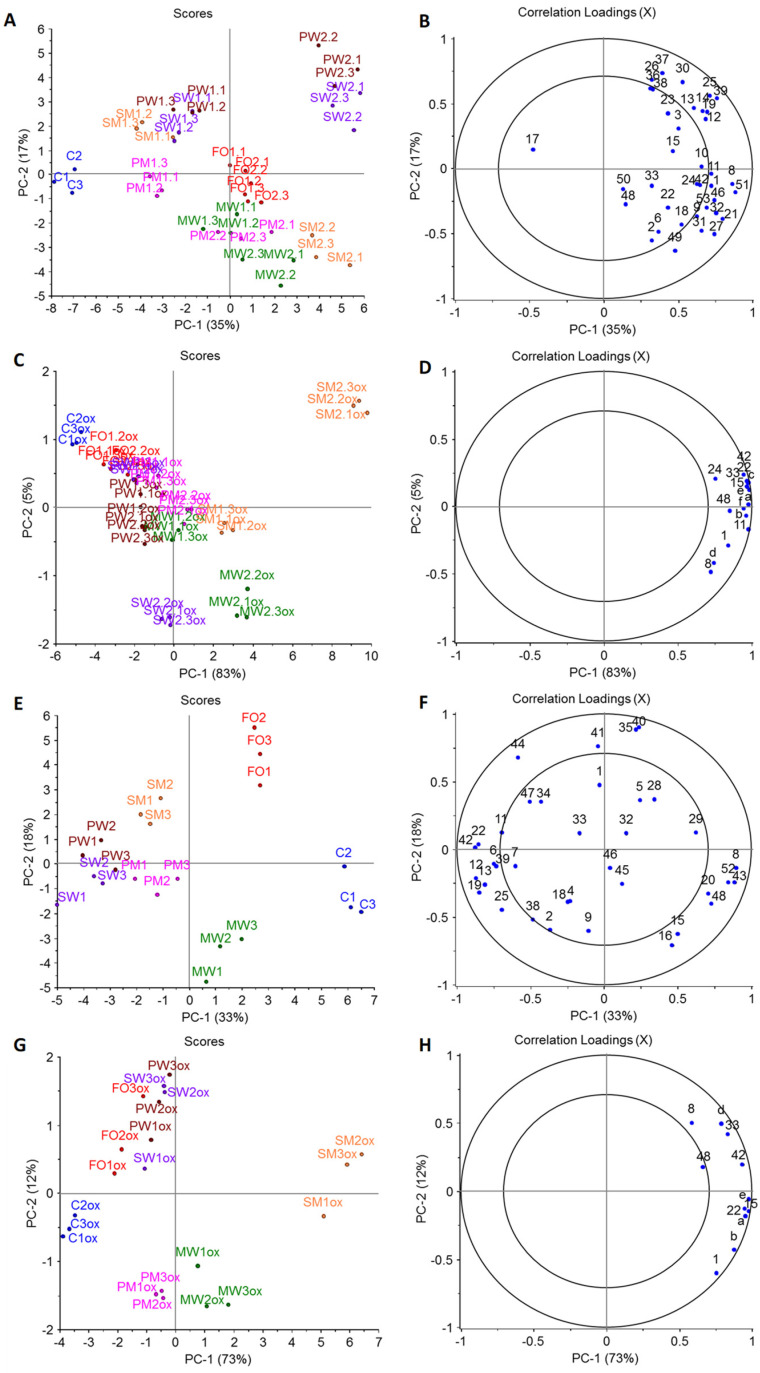
PCAs of identified volatile compounds in cookie (**A**,**B**) and chocolate (**E**,**F**) food model and PCAs of selected volatile oxidation indicators in stored cookie (**C**,**D**) and chocolate (**G**,**H**) food model (n = 3). In scores plots: C = control, FO = fortified with non-encapsulated fish oil, spray-dried emulsion with M = maltodextrin, W = WPC = whey protein concentrate, SF = sunflower protein, S = soy protein, or P = pea protein as wall material, first number indicates concentration (1 lower and 2 higher concentration of fish oil) and last replicate, ox = stored samples; in loading plots: **1** = acetaldehyde, **2** = ethanol, **3** = propanal, **4** = methyl ester of acetic acid, **5** = 2-methylpropanal, **6** = 2,3-butandione, **7** = 2-butanone, **8** = acetic acid, **9** = 2,3-butanediol, **10** = 2-pentanone, **11** = pentanal, **12** = 2-methylhetane, **13** = 4-methylheptane, **14** = 2,4-diemetylhexane, **15** = propanoic acid, **16** = lactic acid, **17** = 1,2-butanediol, **18** = pyridine, **19** = 2,4-dimethylheptane, **20** = 2-methylpropanoic acid, **21** = 2-hexanone, **22** = hexanal, **23** = 2,4-dimethyl-1-heptene, **24** = butanoic acid, **25** = 4-methyloctane, **26** = nonane, **27** = furfural, **28** = 3-methylbutanoic acid, **29** = 2-methybutanoic acid, **30** = 2,7-dimethyloctane, **31** = 2-furanmethanol, **32** = 2-heptanone, **33** = heptanal, **34** = 2,5-dimethylpyrazine, **35** = α-pinene, **36** = 4-methylnonane, **37** = 2-methylnonane, **38** = 2,5-dimethylnonane, **39** = 4-methyldecane, **40** = 3-carene, **41** = trimethylpyrazine, **42** = hexanoic acid, **43** = D-limonene, **44** = 2-ethyl-3,5-dimethylpyrazine, **45** = tetramethylpyrazine, **46** = 2-nonanone, **47** = linalool, **48** = nonanal, **49** = maltol, **50** = δ-hexalactone, **51** = 2,3-dihydro-2,3-dihydroxy-6-methyl-4*H*-pyran-4-one, **52** = nonanoic acid, **53** = 2-undecane, **a** = 2-ethylfuran, **b** = 1-penten-3-ol, **c** = 2-hexenal (*E*), **d** = 3,5-octadien-2-one (*E,Z*), **e** = 5-ethyl-2(5*H*)-furanone, **f** = 2-nonenal (*E*).

In the PCA model of the volatile profile of the fortified cookies (Figure 3A,B), PC-1 accounted for 35% and PC-2 for 17% of the variation. Replicates of samples grouped together are shown in Figure 3A,C, positioned on the left side, was separated from the fortified samples only associated with 1,2-butanediol, which may be related to better extractability as no volatiles from fish oil were present. 1,2-Butanediol was also present in other samples, but the contribution of other volatiles was greater if samples contained fish oil. FO grouped in the center of PCA related to nonanal and δ-hexalactone (Figure 3B) regardless of concentration, while samples with encapsulated fish oil separated based on concentration. All encapsulated fish oil cookie samples were found further on the right if a higher concentration was added. The samples containing M showed the tendency to be found in the lower half of PCA and the ones only containing proteins as encapsulation material were located on the upper half of PCA, except for SM1, which grouped with SW1 and PW1 in the upper half (Figure 3A). SW2 and PW2 grouped together in the right upper corner and were mainly associated with hydrocarbons (like 4-methyldecane, 4-methylocyane, and 2,4-dimethylheptane), and propanal. PM1, MW1, MW2, PM2, and SM2 were all found in the lower half of the PCA related to maltol, ethanol, 2,3-butanedione, 2,3-butanediol, and Maillard reaction products. MW showed a lower impact of concentration compared with samples with other encapsulation material combinations (Figure 3A). If the PCA model of volatile profiles is compared to PCA models and the descriptive sensory profiles (Figure 2A and Figure 3A), some correlation can be found. In both cases, PM1 and SM1 resembled the C most closely. Both volatiles profiles and descriptive sensory profiles showed high fish oil concentration dependency.

For the chocolate model, 36 volatile compounds were identified (Table 3), of which acetic acid was the most abundant followed by 3-methylbutanoic acid and tetrametylpyranzine. Identified compounds were found in all chocolate models regardless of fortification. The chocolate contained alcohols (e.g., ethanol and 2,3-butanedione), acids (like acetic acid, lactic acid, propanoic acid, 3-methyl, and 2-methylbutanoic acid), and esters (e.g., methyl ester of acetic acid) formed from the fermentation process of cocoa bean [36]. Pyrazines (like 2,5-dimethylpyrazine, trimethylpyrazine, 2-ethyl-3,5-dimethylpyrazine, and tetramethylpyrazine) and aldehydes (e.g., 2-methylpropanal, hexanal, and nonanal) detected were produced during the roasting process [36]. Further, α-pinene, 3-carene, and linalool were identified in chocolate, which are all terpenes previously detected in dark chocolate [36,37].

The PCA model for the chocolate samples (Figure 3E,F), PC-1, accounted for 33% while PC-2 accounted for 18% of the variation. All replicates grouped together and C and FO were located by themselves (Figure 3E). C, FO, and MW were all found on the right side of PC-1. C was related to acetic acid, D-limonene, and nonanoic acid, FO to α-pinene, 3-carene, 2-methylpropanal, and 3-methylbutanoic acid, and MW to propanoic acid and lactic acid. SW, PW, SM, and PM were located on the left side of PC-1 with the ones containing M closer to the center of PCA. SW and PW grouped together and were associated with 2-methylheptane, 2,4-methylheptane, hexanal, hexanoic acid, and 2,3-butanedione (Figure 3F). SM was found on the left upper quadrant and is more related to 2-ethyl-3,5-dimethylpyrazine, linalool, and 2,5-dimethylpyrazine. Compared to SM, PM was located in the left lower quadrant associated with 2-butanone, 4-methyloctane, 2-methylheptane, 2,4-methylheptane, and 2,3-butanedione. Less correlation between volatile profiles and descriptive sensory profiles could be found for the chocolate model than for the cookie one. However, both profiles (Figure 2B and Figure 3E) showed that the control was significant different from any of fortified samples. Therefore, fortification had a significant impact on both profiles for the chocolate model.

Although, in case of cookies most and in case of chocolate all identified volatiles were found in all samples, and an effect of fortification and encapsulation material was seen on the ratio of volatiles in the volatile profiles. In cookies, an effect of concentration of encapsulated fish oil was also demonstrated. However, the differences could not only be associated with the addition of FO and encapsulated fish oil as mostly volatiles from the food matrix were affected. This points to differences in the release most likely caused by compositional and textural changes of the food model matrix through fortification, also noted in the sensory analysis.

### 2.4. Storage Test of Food Models

**Figure A1:**
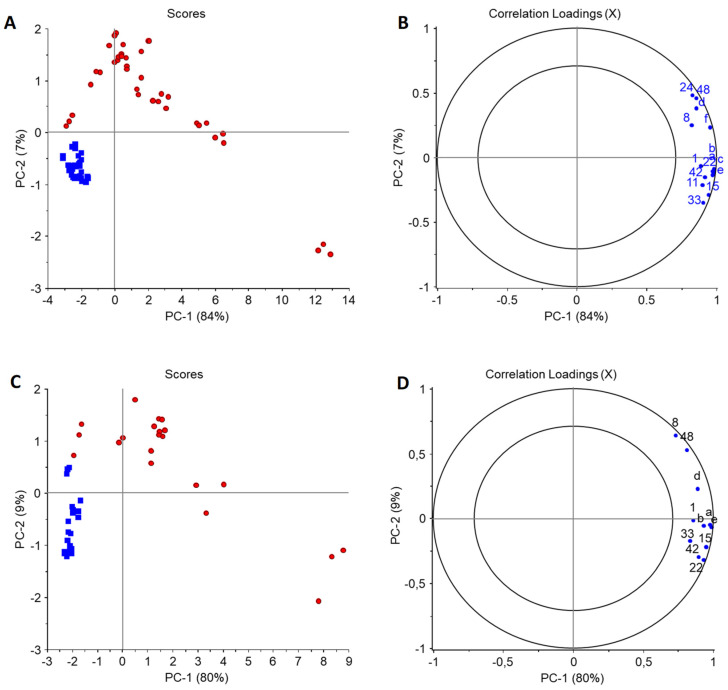
PCAs of selected volatile oxidation indicators in fresh and stored cookie (**A**,**B**) and chocolate (**C**,**D**) food model (n = 3). In scores plots: blue = fresh, red = stored; in loadings plots: **3** = propanal, **a** = 2-ethylfuran, **8** = acetic acid, **b** = 1-penten-3-ol, **11** = pentanal, **15** = propanoic acid, **22** = hexanal, **24** = butanoic acid, c = 2-hexenal (*E*), **33** = heptanal, **42** = hexanoic acid, **d** = 3,5-octadien-2- one (*E,Z*), **48** = nonanal, **e** = 5-ethyl-2(5H)-furanone, **f** = 2-nonenal (*E*).

Volatile profiles of stored food models (6 months, RT) were compared to volatile profiles of fresh ones, and in the case of cookies and chocolate 15 and 11 oxidation indicators were determined, respectively. Oxidation indicators only detected in samples containing fish oil were propanal, 2-ethylfuran, 1-penten-3-ol, 2-hexenal (*E*), 3,5-octadien-2-one (*E,E*), 5-ethyl-2(5*H*)-furanone, and 2-nonenal (*E*), of which 2-hexenal (*E*) and 2-nonenal (*E*) were only detected in the cookie model. All compounds are common volatile oxidation products for *n*–3-rich fish oil [13,26]. Other selected oxidation indicators were acetic acid, pentanal, propanoic acid, hexanal, butanoic acid, heptanal, hexanoic acid, and nonanal, which were also present in C and could also be formed from other lipids present. Pentanal and butanoic acid were not detected from chocolate. In the PCA models of oxidation indicators in fresh and stored samples, PC-1 explained 84% and 80% of total variance for the cookies and chocolates, respectively (Appendix B; Figure A1). For both food models, all fresh samples were grouped together on the left side of PCAs with only the stored C being close to fresh samples. The models were heavily influenced by the fact that oxidation indicators originating from fish oil were only detected in stored and not in fresh samples, except propanal in the cookie model. This clearly indicated that all models oxidized during storage. For determination of the effect of encapsulation material on lipid oxidation during storage, PCAs only taking the stored samples into account were conducted (Figure 3).

The loadings of oxidation indicators in the PCAs of the oxidized samples for both cookies and chocolate were all on positive site of PC-1 (Figure 3D,H), which means the further right a sample is located on PC-1, the more oxidized it is. In the case of cookies, all replicates of C, FO, SW, and PW were located on the negative side of PC-1 (Figure 3C) with C being, as expected, the furthest left and least oxidized. MW and PM displayed a clear concentration dependency. All cookies with higher concentrations were further right on PC-1 compared with lower concentrations using the same encapsulation material. A similar effect of concentration was also seen for oxidative stability of bread fortified with microencapsulated *n*–3 PUFAs powder as higher concentration promoted oxidation [28]. The effect was lowest for PW. In the case of SM, both concentrations were found on the positive side of PC-1, and it was clearly the most oxidized of all cookie models. PC-2 displayed some differences in the formation of lipid oxidation indicators (Figure 3D), which can be related to the extent of oxidation as certain compounds are formed earlier than others. In the case of chocolate, C was also located the farthest on the left side of PC-1 (Figure 3G). Further, FO, SW, PW, and PM were found on the negative side of PC-1, while MW and SM were found on the positive side of PC-1. FO, SW, PW, and SM correlated more to acetic acid, 3,5-octadien-2-one (*E,Z*), heptanal, nonanal, and hexanoic acid (Figure 3H), as they were located on the positive side of PC-2 (Figure 3G). However, PM and MW, located on the negative side of PC-2, were more correlated to propanal and 1-penten-3-ol (Figure 3H).

As expected, for both food models, C was the least oxidized. In the case of cookies, the oxidation order from least to most oxidized was FO, SW, PW, PM, MW, and SM for the lower and FO, PW, SW, PM, MW, and SM for the higher concentration. In the case of chocolate oxidation order from least to most oxidized was FO, SW/PW, PW/SW, PM, MW, and SM. The order was similar in both food models. The oxidative stability of food models containing encapsulated material was similar to the oxidative stability of SDEMs used. In both cases, the samples containing W were more oxidative stable than samples containing M. However, it cannot be concluded that oxidative stability is fully dependent on oxidative stability of the SDEMs as PM performed better in food models as expected based on oxidative stability of the SDEMs and MW performed worse than expected. Surprisingly, the highest oxidative stability in both fortified food models was seen if they contained FO. High oxidative stability was also seen for fish oil-fortified nutrition bars compared with the control based on hexanal and propanal levels, and peroxide value after 10 weeks of storage [27]. The effect was mainly attributed to the addition of tocopherols in the fish oil and the use of soy products containing isoflavones with antioxidant activity. However, compared with this study, no encapsulated fish oil was studied. Encapsulation clearly improved the oxidative stability of the fish oil, as seen in Section 2.1. Yet, it could be that the SDEMs contain more prooxidants, e.g., already formed hydroperoxides from processing or metals introduced during processing than the FO. Spray-drying could also have a negative effect on antioxidants content as spray-drying has been shown to decrease the tocopherol content in oil [38]. Further, the non-polar fish oil might be better incorporated in high fat food models than the more polar encapsulated oil. The fish oil-fortified nutrition bars with high oxidative stability studied by Hughes et al. [27] also contained significantly high fat content emulsifying properties based on the ingredient list reported. This raises the question of whether encapsulation is always necessary in terms of storage stability if fish oil is incorporated in high fat food models.

## 3. Materials and Methods

### 3.1. Materials

#### 3.1.1. Spray-Dried Emulsions (SDEMs)

FO, commercial cod liver oil (Möller’s Orkla, Oslo, Norway), was encapsulated by spray-drying [14] using either M (DE 14—22, Edpol Food & Innovation company, Lomza, Poland), and W (WPC 80, Ostrowia Company, Warszawa, Poland) (1:1; MW), SF (sunflower protein, Barentz Food & Nutrition, Hoofddorp, Netherlands) and W (1:1; SFW), S (soy protein, Barentz Food & Nutrition, Netherlands) and W (1:1; SW), P (pea protein, Roquette, Lestrem, France) and W (1:1; PW), SF and M (1:1; SFM), S and M (1:1; SM), or P and M (1:1; PM) as coating material combinations (Table 4).

Aqueous emulsions of the core (FO; 10%) and wall material solution (10% of wall material combination (MW, SFW, SW, PW, SFM, SM, PM)) were formed using Thermomix (Vorwerk, Wuppertal, Germany) operated at 9000 rpm for 120 s at 40 °C. The emulsions were homogenized at 240 bar (I step) and 40 bar (II step) using a high-pressure laboratory valve homogenizer (Panda 2K, GEA Niro Soavi, Parma, Italy). The emulsions were pumped into a pilot plant spray dryer (A/S Niro Atomizer, Copenhagen, Denmark; spraying mechanism—disc with a 110 mm diameter, 6400 rpm number of revolutions) chamber at a constant feeding speed of 77 mL/min. The airflow rate was approx. 400 kg/h, and the temperatures of inlet and outlet were 130 °C and 90 °C, respectively.

#### 3.1.2. Food Models: Shortbread Cookie and Dark Chocolate

The formula of shortbread (*w*/*w*) was 34.5% of butter (Valio Ltd., Helsinki, Finland), 50.4% of wheat flour (Myllyn Paras Finland Oy, Hyvinkaa, Finland), and 15.2% of sugar (granulated sugar, Rainbow, Sucros Oy, Säkylä, Finland), purchased locally from the grocery store. Oil or powder was added so that it replaced flour.

Butter (of room temperature) and sugar were mixed followed by gradual addition of wheat flour. Two concentrations of fish oil (as such or encapsulated MW, SW, PW, SM, or PM) were added to the shortbread cookies to reach 40 mg of the sum of EPA or DHA per 100 g and per 100 kcal (claim: source of *n*–3 fatty acids; (1), or 80 mg of the sum of EPA or DHA per 100 g and per 100 kcal (claim: high in *n*–3 fatty acids; (2). Additions were calculated based on the total oil content of encapsulated oil and the fatty acid composition of FO (see Section 2.1). Additionally, for sensory training, cookies were made with 0.5, 1.5, 2.5, and 5 times the health claim of 40 mg of the sum of EPA or DHA per 100 g and per 100 kcal using fish oil. A fish oil-free control sample was also included. The dough was rolled into balls and then shaped into cookies (approx. 4 cm in diameter) and baked at 175 °C for 14 min. After the baking the average weight of one cookie was 12.9 g (standard deviation 0.56 g).

The dark chocolate couverture (70% Arriba cacao, Ecuador; Lidl Stiftung & Co. KG, Neckarsulm Germany) was cut and heated to 45 °C and kept at this temperature for 15 min to ensure complete melting. FO or encapsulated fish oil (MW, SW, PW, SM, or PM) was mixed with the chocolate mass at 35 °C for 2 min. A three-stage tempering was applied: 40 to 45 °C for 10 min, 26 to 28 °C for 5 min, and 30 to 32 °C for 10 min. The tempered mass was immediately filled into silicone molds (115 mm × 77 mm × 9 mm, length × width × height) and kept for 12 h at 4 ± 1 °C. The FO and encapsulated fish oil were added to chocolate concentrations to reach 60 mg of the sum of EPA or DHA per 100 g and per 100 kcal (claim: source of *n*–3 fatty acids). Additions were calculated based on the total oil content of encapsulated oil and the fatty acid composition of FO (see Section 2.1). As a control, chocolate without fish oil was prepared.

#### 3.1.3. Reagents and Chemicals

Commercial, analytical grade chemicals were used: 2-propanal from Honeywell International Inc. (Riedel de Häen, Seelze, Germany), n-hexane, methanol, and chloroform from Sigma-Aldrich (Saint Louis, MO, USA), potassium chloride from VWR Chemicals (Leuven, Belgium), potassium carbonate and acetyl chloride from Sigma-Aldrich (Saint Louis, MO, USA). Heptadecanoic acid (Larodan, Stockholm, Sweden) was used as internal standard and 68D and GLC-490 (Nu-Check-Prep, Inc., Elysian, MN, USA) and Supelco 37 Component FAME Mix (Supelco, Inc., Bellefonte, PA, USA) as external standards for fatty acid analysis. For sensory analyses, 2% sucrose (sucrose purity 99%, Alfa Aesar, Karlsruhe, Germany) and 0.07% caffeine solutions (caffeine purity 99%, Alfa Aesar, Karlsruhe, Germany) were prepared. Other reference samples for evaluations, purchased from a local grocery store, are listed in Table 5.

### 3.2. Methods for Characterization of SDEMs

#### 3.2.1. SEM

The powder was attached to the microscope state using two-sided adhesive tape, mounted on SEM tubs, and coated with palladium in a sputter coater. Samples were analyzed using SEM Quanta 200 (FEI Company, Hillsboro, OR, USA) operating an accelerating voltage of 30 kV and 400× magnifications.

#### 3.2.2. Total and Surface Oil Content and EE

Lipids were extracted with n-hexane or a chloroform/methanol mixture (2:1, *v*/*v*) for surface and total oil, respectively, and weighted according to the method by Takeungwongtrakul et al. [39], also previously described in Damerau et al. [13]. EE was calculated by using the following equation:

EE (%) = (Total oil − Surface oil)/Total oil × 100(1)

#### 3.2.3. Fatty Acid Profiles

Fatty acid methyl esters by GC-FID were analyzed as previously described (Damerau et al., 2022). Encapsulated lipids were extracted with n-hexane/2-propanal mixture (3:1, *v*/*v*) after re-suspending the sample in 0.8% potassium chloride in MQ-water. Methanolic hydrogen chloride was used in methylation [40]. A Shimadzu GC-2030 with an AOC-20i auto injector and an FID (Shimadzu corporation, Kyoto, Japan) equipped with a DB-23 (60 m × 0.25 mm i.d., liquid film 0.25 μm, Agilent Technologies, J.W. Scientific, Santa Clara, CA, USA) column were used. GC conditions: helium flow 1.4 mL/min; 130 °C held 1 min, 6.5 °C/min to 170 °C, 2.75 °C/min to 205 °C, held for 18 min, 30 °C/min to 230 °C and held for 2 min. The peaks were identified by using the external standards, and FAs quantified using the internal standard and correction factors determined with the external standard mixtures.

#### 3.2.4. OSI

A 743 Rancimat (Metrom, Zofingen, Switzerland) eight-channel oxidative stability instrument was used. Samples (2.5 g each) were placed in a reaction vessel in a thermostatic electric heating block. The temperature was set at 110 °C and an airflow rate at 20 L/h. The OSI was expressed as hours [13].

### 3.3. Sensory Evaluation

The panelists, 10 women and 2 men, were recruited among the staff and the students of the University of Turku. As an inclusion criterion, all panelists had prior experience in sensory evaluation. They were familiarized with the samples and sensory attributes during two 1 h training sessions. The generic descriptive method was used to determine the effects of SDEMs and oil concentrations. Two odor attributes, five flavor attributes, and six texture attributes were selected (Table 5). Panelists were tested between and after all sessions for producing reliable and reproducible results.

The sensory evaluations were performed by ISO 8589 standard. The samples, 6.5 g of cookie or 4.0 g piece of chocolate, were served in glass bowls at room temperature and labelled with three-digit random codes in an order that followed a Latin Squared design. Samples were evaluated in duplicate; cookies in four and chocolates in two sessions. The assessors were instructed to take a sample in the mouth, chew and rotate it in the mouth, and give their first impression. With mouthfeel and texture attributes, continued chewing of the sample to evaluate an after-effect was instructed. The intensities of the attributes (Table 5) were evaluated on a scale of 0–10 (0; no attribute observed, 10; strong attribute observed) with the help of reference samples. The assessors were instructed to clean their mouths by drinking activated carbon-filtered water and chewing an unsalted water biscuit (Carr’s Table Water biscuit, United Biscuits, London, UK) between samples. The data were collected by the Compusense Cloud (version 8.4, Compusense Inc., Guelph, ON, Canada).

### 3.4. Storage Test

The cookie and chocolate models were stored at room temperature for 6 months and packaged in food-grade plastic containers. Fresh and stored samples were kept under nitrogen at −80 °C until analysis. 

#### Analysis of Volatiles

VSOPs of SDEMs were analyzed using HS-SPME-GC-MS as described by Damerau et al. [41]. 0.5 g ± 0.005 g of SDEM, 1 g ± 0.01 g of the crushed cookie models, and 1 g ± 0.01 g of finely chopped chocolate models were weighed in triplicate in 20 mL headspace vials and flushed with nitrogen. Volatiles were extracted using HS-SPME using TriPlus RSH autosampler (Thermo Scientific, Reinach, Switzerland) equipped with a DVB/CAR/PDMS-fiber (50/30 μm film thickness; Supelco, Bellefonte, PA, USA). Extraction conditions: agitation speed 250 rpm, incubation 50 °C for 20 min, extraction 50 °C for 30 min, and desorption 240 °C for 6 min. Extracted volatiles were analyzed with TRACE 1310 GC (Thermo Scientific Reinach, Switzerland) equipped with a SPB^®^-624 capillary column (60 m × 0.25 mm × 1.4 μm, Supelco, Bellefonte, PA, USA) and coupled with an ISQ 7000 MS detector (Thermo Scientific, Reinach, Switzerland). GC-MS conditions: helium flow 1.4 mL/min; oven at 40 °C held 6 min, 5 °C/min to 220 °C and held for 10 min; EI mode 70 eV and scan range 40 to 300 amu. Compounds were identified by the NIST MS Search library (version 2.3. National Institute of Standards and Technology, Gaithersburg, MD, USA) and by comparing retention times and MS spectra of standards. 

### 3.5. Statistical Analysis 

PanelCheck (version 1.4.2, Nofima, Tromsø, Norway) was used to evaluate the performance of the panel and assessors. Reproducibility and discriminability of assessors and agreement of the panel were analyzed using univariate (p-MSE, F&p, MSE, correlation, and eggshell plot) and multivariate methods (Tucker-1 and Manhattan plots) as described by Tomic et al. [42]. 

An overview of the sensory profile of cookies and chocolates was formed with PCA using averaged data over assessors and replicates by Unscrambler^®^ X version 11.0 (Camo Analytics AS., Oslo, Norway). The data were autoscaled, and full cross-validation and singular value decomposition were used. PCA was also applied to averaged peak area data to determine the correlation of volatiles and samples at different time points. Data were mean-centered and weighed (1/sdev). 

The data of the descriptive analysis were analyzed by ANOVA for each attribute. Between subject factors used for chocolate were plant proteins (S and P) and wall materials (M and W) and for cookies the two above-mentioned as well as the oil concentration (1.5% and 3.0%). Pairwise comparisons were calculated with either Bonferroni adjustment or Tukey’s HSD post hoc test. Statistical tests were performed with IBM SPSS Statistics 27 (IBM Corporation, Armonk, NY, USA). The criterion of statistical significance was *p* < 0.05.

## 4. Conclusions

Encapsulation by spray-drying using pea, soy, and sunflower protein in combination with either whey protein concentrate or maltodextrin increased oxidative stability of fish oil. The best protection was achieved with a combination of pea or soy protein with whey protein concentrate. The fatty acid composition of the oil was not affected by the encapsulation process. The most promising formulations were successfully used for the fortification of shortbread cookie and dark chocolate food models, which were studied as such and during storage. 

The sensory panel detected differences between fortified food models and controls regardless of formulation. The main attributes for the distinction were fishy flavor and odor. Increasing the oil content in cookies had a negative impact on sensory perception. For both food models, fortification using fish oil encapsulated with pea protein and maltodextrin showed the closest resemblance to control. Based on sensory perception, mainly textural changes, the cookies were a more promising model than chocolate for fortification. 

Volatiles profiles could be differentiated based on fortification and encapsulation material for both tested food models. This was mainly due to compositional and textural changes affecting the release of volatiles. Six months storage at room temperature revealed high oxidative stability for food models fortified with fish oil encapsulated with pea or soy protein with whey protein concentrate, similar as for spray-dried emulsion themselves based on volatile oxidation products. Increasing oil content had a negative impact on the oxidative stability. While as hypothesized some of the plant protein containing formulations showed good potential, fortification using non-encapsulated fish oil was surprisingly promising as well based on sensory perception, volatile profiles, and oxidative stability for the high-fat food models used in this study. Therefore, the most ability for fortification was seen for pea protein formulations and direct addition of fish oil using the cookie model. However, further research studying sensory perception during storage and effect on fortification in less fatty food matrices is needed to evaluate the full potential of encapsulation with pea protein. Further, analysis by gas chromatography-olfactometry would be useful to study odor activity and to allow better correlation of sensory and volatile data.

## Figures and Tables

**Table 1:** Total and surface oil content, encapsulation efficiency, oxidative stability index, and total peak area of selected volatile oxidation indicators of spray-dried emulsions.

Spray-Dried Emulsion *	Total Oil (%)	Surface Oil (%)	Encapsulation Efficiency (%)	OSI (h) **	The Total Peak Area of Oxidation Indicators ***
**MW**	40.66 ± 0.39	13.94 ± 0.08	65.70 ± 0.52	12.67 ± 0.08	18.7 ± 1.2
**SFW**	44.18 ± 1.06	13.39 ± 1.79	69.64 ± 4.78	8.31 ± 0.35	47.1 ± 0.9
**SW**	44.14 ± 1.59	14.62 ± 1.57	66.79 ± 4.75	12.79 ± 0.27	17.1 ± 1.5
**PW**	41.22 ± 0.51	15.90 ± 0.53	61.06 ± 0.93	15.66 ± 0.21	15.9 ± 1.6
**SFM**	40.70 ±0.06	15.87 ± 0.44	61.06 ± 1.04	3.92 ± 0.22	128.6 ± 7.6
**SM**	38.62 ± 0.46	14.03 ± 0.56	63.66 ± 1.87	6.38 ± 0.07	44.8 ± 2.5
**PM**	40.63 ± 0.32	17.35 ± 1.65	57.27 ± 4.39	11.47 ± 0.16	27.7 ± 1.8

* Spray-dried emulsion with M = maltodextrin, W = WPC = whey protein concentrate, SF = sunflower protein, S = soy protein, or P = pea protein as wall material. ** OSI—oxidative stability index at 110 °C, for non-encapsulated oil was 1.36 ± 0.14 h. *** Total peak area of selected volatile oxidation indicators (propanal, 2-ethylfuran, hexanal, 2-hexenal (*E*), 2-pentylfuran, 3,5-octadien-2-one (*E,Z/E,E*), nonanal) in counts per s per 10^5^ analyzed by headspace solid-phase microextraction with gas chromatography with mass spectrometer detection.

**Table 2:** Fatty acid composition (% of total fatty acids) of non-encapsulated and encapsulated fish oil, including sums of saturated fatty acids (Σ SFA), monounsaturated fatty acids (Σ MUFA), polyunsaturated fatty acids (Σ PUFA), and *n–*3 and *n–*6 fatty acids.

Fatty Acid	Fish Oil	MW *	SFW *	SW *	PW *	SFM *	SM *	PM *
**14:0**	4.28 ± 0.01	4.36 ± 0.11	4.33 ± 0.01	4.40 ± 0.13	4.35 ± 0.05	4.27 ± 0.05	4.20 ± 0.14	4.21 ± 0.04
**16:0**	10.70 ± 0.01	11.24 ± 0.15	11.26 ± 0.16	11.30 ± 0.11	11.40 ± 0.10	10.75 ± 0.03	10.90 ± 0.04	10.89 ± 0.02
**16:1 (*n*–7)**	10.73 ± 0.10	10.32 ± 0.09	10.41 ± 0.18	10.31 ± 0.06	10.08 ± 0.18	10.63 ± 0.12	10.60 ± 0.08	10.39 ± 0.09
**18:0**	2.22 ± 0.04	2.44 ± 0.03	2.47 ± 0.03	2.47 ± 0.01	2.55 ± 0.03	2.29 ± 0.01	2.32 ± 0.01	2.33 ± 0.01
**18:1 (*n*–9)**	16.17 ± 0.07	16.10 ± 0.14	16.22 ± 0.15	16.14 ± 0.10	16.69 ± 0.14	16.17 ± 0.03	16.03 ± 0.07	16.69 ± 0.09
**18:1 (*n*–7)**	5.38 ± 0.02	5.17 ± 0.10	5.14 ± 0.06	5.19 ± 0.01	5.12 ± 0.02	5.35 ± 0.01	5.35 ± 0.05	5.25 ± 0.02
**18:2 (*n*–6)**	2.53 ± 0.04	2.58 ± 0.03	2.83 ± 0.04	3.09 ± 0.03	3.32 ± 0.30	2.75 ± 0.04	3.23 ± 0.02	3.02 ± 0.12
**18:3 (*n*–3)**	0.98 ± 0.02	1.23 ± 0.04	1.08 ± 0.07	1.02 ± 0.07	1.28 ± 0.01	1.00 ± 0.01	1.05 ± 0.03	1.33 ± 0.02
**20:1 (*n*–9)**	14.58 ± 0.02	14.61 ± 0.55	14.50 ± 0.65	14.41 ± 0.71	13.79 ± 0.10	14.56 ± 0.05	14.22 ± 0.11	14.09 ± 0.03
**20:5 (*n*–3)**	10.34 ± 0.03	10.06 ± 0.14	10.03 ± 0.14	9.96 ± 0.09	9.77 ± 0.09	10.22 ± 0.01	10.17 ± 0.04	9.98 ± 0.04
**22:5 (*n*–3)**	1.37 ± 0.00	1.34 ± 0.02	1.33 ± 0.01	1.32 ± 0.01	1.28 ± 0.00	1.36 ± 0.01	1.34 ± 0.02	1.30 ± 0.00
**22:6 (*n*–3)**	14.69 ± 0.05	14.56 ± 0.19	14.46 ± 0.19	14.29 ± 0.10	14.35 ± 0.25	14.57 ± 0.02	14.31 ± 0.08	14.45 ± 0.16
Other **	6.03 ± 0.03	5.98 ± 0.15	5.96 ± 0.22	6.09 ± 0.08	6.00 ± 0.03	6.09 ± 0.07	6.12 ± 0.04	6.04 ± 0.08
**Σ SFA**	17.57 ± 0.05	18.41 ± 0.15	18.43 ± 0.20	18.54 ± 0.24	18.67 ± 0.17	17.67 ± 0.08	17.78 ± 0.19	17.81 ± 0.07
**Σ MUFA**	48.34 ± 0.04	47.70 ± 0.46	47.68 ± 0.43	47.54 ± 0.57	47.15 ± 0.36	48.20 ± 0.14	47.65 ± 0.22	47.86 ± 0.14
**Σ PUFA**	31.42 ± 0.07	31.23 ± 0.33	31.28 ± 0.36	31.22 ± 0.30	31.51 ± 0.33	31.44 ± 0.06	31.65 ± 0.14	31.59 ± 0.13
**Σ *n*–3**	27.64 ± 0.07	27.44 ± 0.33	27.17 ± 0.30	26.85 ± 0.26	26.94 ± 0.37	27.42 ± 0.04	27.14 ± 0.14	27.31 ± 0.21
**Σ *n*–6**	3.78 ± 0.02	3.79 ± 0.02	4.11 ± 0.06	4.37 ± 0.04	4.57 ± 0.08	4.02 ± 0.06	4.51 ± 0.05	4.28 ± 0.12

* Spray-dried emulsion with M = maltodextrin, W = WPC = whey protein concentrate, SF = sunflower protein, S = soy protein, or P = pea protein as wall material. ** Other fatty acids include: 12:0, 14:1 (*n–*5), 15:0, 15:1, 18:3 (*n*–6), 20:0, 20:2 (*n*–6), 20:3 (*n*–6), 20:4 (*n*–6), 20:3 (*n*–3), 22:0, 22:1 (*n*–9), 22:3 (*n*–3), 22:4 (*n*–6), 24:1 (*n*–9), trans-18:1 (*n*–9), and trans-18:2 (*n*–6); all under 1%.

**Table 3:** Retention times, match with NIST library, and main ions in the mass spectra of 53 identified volatile compounds analyzed by headspace solid-phase microextraction with gas chromatography with mass spectrometer detection from non-stored shortbread cookie and dark chocolate models fortified with fish oil.

	Compound	RT (min)	Match with NIST20	Main Ions (m/z)	Cookie Model **	Chocolate Model **
1	acetaldehyde	6.63	861	42, 43, 44	x	x
2	ethanol	9.21	900	43, 45	xx	x
3	propanal *	10.20	943	42, 43, 57, 58	x	
4	acetic acid, methyl ester	11.44	809	43, 59, 74		x
5	2-methylpropanal	13.23	813	43, 57, 72		x
6	2,3-butanedione	15.22	953	42, 69, 86	xx	x
7	2-butanone	15.69	906	43, 57, 72		x
8	acetic acid	18.29	966	43, 55, 60	xxx	xxx
9	2,3-butanediol	19.84	917	45, 57, 75, 90	xx	xx
10	2-pentanone	20.51	850	43, 58, 71, 86	xx	
11	pentanal	20.83	856	44, 57, 58	xx	xx
12	2-methylheptane	22.21	938	43, 57, 70	xx	x
13	4-methylheptane	22.33	910	43, 55, 70	xx	x
14	2,4-dimetylhexane	22.66	859	43, 57, 85, 114	x	
15	propanoic acid	22.95	817	45, 57, 74	x	x
16	lactic acid	23.10	797	45, 56, 74, 86		xx
17	1,2-butanediol	23.55	783	45, 59, 73, 89	x	
18	pyridine	23.69	913	52, 60, 79	xx	xx
19	2,4-dimethylheptane	25.11	937	43, 57, 71, 85	xxx	xx
20	2-methylpropanoic acid	25.69	910	41, 43, 73, 88, 113		xx
21	2-hexanone	25.79	754	43, 58, 100	xx	
22	hexanal	26.08	975	41, 44, 56, 67, 72	xx	xx
23	2,4-dimethyl-1-heptene	26.32	804	43, 55, 70, 83, 126	x	
24	butanoic acid	27.00	852	60, 73, 88	x	
25	4-methyoctane	27.08	956	43, 57, 71, 85, 128	xx	xx
26	nonane	28.70	913	43,57, 85, 99, 128	x	
27	furfural	29.06	812	39, 50, 67, 95, 96	xx	
28	3-methylbutanoic acid	29.49	932	43, 60, 69, 87, 101		xxx
29	2-methybutanoic acid	29.80	907	41, 57, 74, 87, 101		xx
30	2,7-dimethyloctane	30.00	897	43, 57, 71, 99	x	
31	2-furanmethanol	30.19	864	41, 53, 69, 81, 98	xx	
32	2-heptanone	30.44	931	43, 58, 71, 99, 114	xxx	xx
33	heptanal	30.83	891	44, 55, 70, 81, 96, 113	xx	xx
34	2,5-dimethylpyrazine	31.27	705	42, 52, 66, 81, 108		x
35	α-pinene	31.28	856	53, 67, 77, 93, 105, 121, 136		xx
36	4-methylnonane	31.49	898	43, 57, 71, 98, 112, 142	x	
37	2-methylnonane	31.53	888	43, 57, 71, 85, 98, 127	xx	
38	2,5-dimethylnonane	33.84	899	57, 71, 85, 114, 156	xx	x
39	4-methyldecane	32.34	892	43, 57, 71, 85, 112	xx	xx
40	3-carene	34.57	832	41, 53, 77, 93, 121, 136		xx
41	trimethylpyrazine	34.93	762	42, 81, 94, 122		xx
42	hexanoic acid	35.11	831	41, 55, 60, 73, 87	xx	xx
43	D-limonene	35.36	802	53, 68, 79, 93, 121		xx
44	2-ethyl-3,5-dimethylpyrazine	37.77	712	42, 54, 80, 108, 135		x
45	tetramethylpyrazine	38.04	877	42, 54, 64, 80, 95, 136		xx
46	2-nonanone	38.63	935	43, 58, 71, 85, 99, 142	xxx	x
47	linalool	38.77	725	41, 55, 71, 80, 93, 121		xx
48	nonanal	39.04	866	41, 57, 70, 82, 98, 114	xx	xx
49	Maltol *	41.29	928	43, 55, 71, 97, 126	xx	
50	δ-hexalactone	42.01	802	42, 55, 70, 99, 114	xx	
51	2,3-dihydro-2,3-dihydroxy-6-methyl-4*H*-pyran-4-one	42.63	894	43, 55, 72, 85, 101, 115, 126, 144	xx	
52	nonanoic acid	45.62	726	41, 60, 73, 83, 98, 115, 158		xx
53	2-undecane	45.86	877	43, 58, 71, 112, 170	xx	

* Volatile compounds not identified in controls (not containing fish oil). ** x = minor compound (peak area < 5 × 10^6^), xx = main compound (peak area > 5 × 10^6^), xxx = major compound (peak area > 5 × 10^7^).

**Table 4:** Sample codes and dry matter composition of spray-dried emulsion and non-encapsulated fish oil.

Sample Code	Cod Liver Oil (%)	Plant Protein (%)	Maltodextrin (%)	Whey Protein Concentrate (%)
MW	50	-	25	25
SFW	50	25/sunflower	-	25
SW	50	25/soy	-	25
PW	50	25/pea	-	25
SFM	50	25/sunflower	25	-
SM	50	25/soy	25	-
PM	50	25/pea	25	-
FO	100	-	-	.

**Table 5:** Sensory attributes evaluated by general descriptive analysis.

Attribute	Description	Reference Sample *	Intensity of Reference **
Fishy odor	Typical odor of fish	-	-
Odor intensity	Total intensity of observed odor	-	-
Sweetness	Perceived sweetness	2% sucrose	4
Bitterness	Perceived bitterness	0.07% caffeine	4
Oily flavor	Oily or fatty flavor	Rapeseed oil	8
Fishy flavor	Typical flavor of fish	-	-
Flavor intensity	Total intensity of observed flavor	-	-
Softness	Softness of the sample during biting	Cookie: shortbread cookies	8
		Chocolates: dark chocolate	4
Moistness (only for cookie)	The feeling of moisture when chewing.	Oat cookie	3
Crumbliness	The sample crumbles into small pieces during chewing.	Cookie: oat cookie	6
		Chocolates: dark chocolate	4
Melting mouthfeel (only for chocolate)	Melting sensation when chewing the sample.	Dark milk chocolate	7
Oily mouthfeel	Oily mouthfeel	Butter	8
Aftertaste	The intensity of taste and flavor after swallowing.	-	-

* Sucrose (purity 99%, Alfa Aesar, Karlsruhe, Germany), caffeine (purity 99%, Alfa Aesar, Karlsruhe, Germany), rapeseed oil (Rainbow, Princes Foods B.V., Rotterdam, Netherlands), butter (Valio Ltd., Helsinki, Finland), shortbreads (Pirkka Eleonora, Kesko Corporation, Helsinki, Finland), oat cookie (Pirkka, Kesko Corporation, Helsinki, Finland), dark chocolate (Karl Fazer 47%, Fazer Confectionery Ltd., Helsinki, Finland), and dark milk chocolate (Marabou Darkmilk Original, Mondelez Finland Ltd., Vantaa, Finland). ** Line scale from 0 (no attribute) to 10 (strong attribute). Cookies and chocolates had different reference samples for softness and crumbliness. For either attribute, the first intensity is for the cookie reference and the latter is for the chocolate reference.

## Data Availability

Data of the chemical and physical analyses is available from the corresponding authors upon reasonable request. Sensory data of the individual panelists is not available to protect the privacy of the panelists.

## Appendix A

**Table A1:** Mean intensities and standard deviations (in brackets) of attributes in the descriptive analysis of the dark chocolate models. The statistical differences between the samples are marked with letters a-f (Tukey’s HSD test, *p* < 0.05). The lowest values are marked with letter a, and samples with the same letter on the same row are not different from each other.

	MW **	SW **	PW **	SM **	PM **	FO **	C **
**Fishy odor ***	0.38 (0.56) a	1.85 (2.15) b	0.48 (0.74) a	0.57 (1.19) ab	0.50 (0.84) a	0.46 (0.71) ab	0.64 (1.33) ab
**Odor intensity**	3.10 (2.05) a	3.87 (1.82) a	3.31 (1.80) a	3.29 (1.27) a	3.23 (1.96) a	2.89 (1.73) a	3.50 (1.86) a
**Sweet**	3.22 (1.81) a	3.35 (1.83) a	3.33 (2.36) a	3.35 (1.78) a	3.9 (1.77) a	3.62 (1.92) a	3.41 (1.88) a
**Bitter**	3.26 (1.97) a	3.28 (2.17) a	3.64 (1.69) a	2.97 (1.67) a	3.59 (1.57) a	3.78 (1.96) a	3.37 (1.82) a
**Oily flavor**	3.57 (1.84) a	3.96 (1.93) a	3.89 (2.13) a	3.90 (2.17) a	4.06 (1.89) a	3.52 (2.00) a	4.42 (1.78) a
**Fishy flavor ***	1.98 (2.13) ab	4.87 (3.19) c	2.92 (2.75) b	1.62 (1.81) ab	1.04 (1.41) ab	1.30 (1.61) ab	0.30 (0.64) a
**Flavor intensity**	4.68 (1.69) a	5.91 (1.91) a	5.01 (1.81) a	4.91 (1.81) a	4.70 (1.44) a	4.83 (1.47) a	4.85 (1.59) a
**Aftertaste**	4.24 (2.02) a	5.50 (2.08) a	4.69 (1.78) a	4.36 (2.02) a	4.38 (1.34) a	4.92 (1.67) a	4.65 (1.68) a
**Softness**	5.39 (1.77) a	5.00 (2.04) a	5.55 (2.48) a	5.59 (2.26) a	6.29 (2.25) a	6.08 (2.01) a	6.82 (1.40) a
**Melting ***	5.14 (1.87) a	5.41 (1.82) a	5.48 (2.22) a	5.48 (2.38) a	6.53 (1.83) ab	6.30 (2.05) ab	7.90 (1.30) b
**Crumbly ***	5.77 (2.26) d	5.75 (2.24) d	5.30 (2.31) cd	3.34 (1.51) bc	3.08 (1.64) ab	4.24 (2.53) bcd	1.74 (1.19) a
**Oily mouthfeel**	3.60 (2.07) a	4.53 (2.27) a	3.80 (2.08) a	3.84 (2.51) a	4.40 (2.39) a	4.18 (2.06) a	5.07 (2.46) a

* Fishy odor and flavor, melting texture, and crumbliness were statistically significant (*p* < 0.05), while other attributes had not significant differences (*p* > 0.05) according to ANOVA. ** FO = non-encapsulated fish oil and spry-dried emulsions formulated M = maltodextrin, W = WPC = whey protein concentrate, S = soy protein or P = pea protein as wall material. Scale from 0 (no attribute) to 10 (strong attribute).

**Table A2:** Mean intensities and standard deviations (in brackets) of attributes in the descriptive analysis of the cookies models. The statistical differences between the samples are marked with letters a-f (Tukey’s HSD test, *p* < 0.05). The lowest values are marked with letter a, and samples with the same letter on the same row are not different from each other.

	MW1 **	MW2 **	SW1 **	SW2 **	PW1 **	PW2 **	SM1 **	SM2 **	PM1 **	PM2 **	FO1 **	FO2 **	C **
**Fishy odor**	0.78 (1.11) ab	1.29 (1.58) abc	1.13 (1.31) abc	2.99 (2.51) d	2.14 (2.14) bcd	2.42 (2.30) dc	0.61 (0.79) a	2.3 (1.92) cd	0.49 (0.65) a	0.95 (1.35) abc	0.72 (0.98) ab	0.8 (0.93) ab	0.32 (0.46) a
**Odor intensity**	3.82 (1.90) ab	4.03 (2.17) ab	3.04 (1.67) ab	4.58 (2.27) b	3.32 (1.70) ab	4.01 (2.09) ab	3.86 (1.72) ab	3.81 (1.46) ab	3.81 (1.96) ab	3.23 (1.86) ab	3.71 (1.97) ab	4.36 (2.39) ab	2.54 (1.31) a
**Sweetness ***	3.81 (1.82) a	3.28 (1.93) a	3.56 (1.97) a	3.48 (1.98) a	3.4 (1.95) a	3.5 (1.83) a	3.7 (1.93) a	3.59 (1.77) a	3.53 (2.05) a	3.93 (1.91) a	3.85 (1.56) a	4.07 (2.24) a	3.64 (1.95) a
**Bitterness ***	0.41 (0.63) a	0.62 (0.83) a	0.52 (0.60) a	0.74 (0.85) a	0.48 (0.55) a	0.46 (0.57) a	0.29 (0.41) a	0.68 (0.75) a	0.42 (0.68) a	0.59 (1.12) a	0.49 (0.73) a	0.67 (0.67) a	0.4 (0.57) a
**Oily**	3.05 (2.08) a	3.15 (1.89) a	3.53 (2.24) a	3.51 (2.40) a	3.87 (2.16) a	4.15 (1.88) a	3.59 (2.10) a	3.66 (2.15) a	3.29 (1.92) a	3.69 (1.94) a	3.4 (2.31) a	3.75 (2.32) a	3.31 (1.97) a
**Fishy flavor**	0.93 (1.15) ab	1.99 (1.95) abc	1.77 (2.22) abc	3.46 (2.88) cd	2.39 (2.30) bc	4.86 (2.82) d	0.76 (0.92) ab	3.16 (3.05) cd	0.5 (0.66) a	1.3 (1.84) ab	0.42 (0.82) a	0.47 (0.76) a	0.31 (0.60) a
**Flavor intensity**	3.75 (1.77) ab	3.41 (1.89) a	3.96 (2.17) ab	4.77 (2.49) ab	4.17 (2.04) ab	5.31 (1.88) b	3.42 (1.49) a	4.39 (2.09) ab	3.41 (1.32) a	3.96 (1.90) ab	3.73 (1.71) ab	4.25 (1.83) ab	3.13 (1.95) a
**Soft mouthfeel**	5.44 (2.07) ab	5.21 (2.01) a	6.28 (1.86) ab	6.02 (2.14) ab	6.23 (2.04) ab	5.72 (1.83) ab	6.15 (1.79) ab	5.13 (2.08) a	6.08 (2.00) ab	5.54 (2.14) ab	7.21 (1.60) b	7.3 (1.82) b	6.1 (1.98) ab
**Moist mouthfeel ***	3.97 (1.67) a	3.6 (1.84) a	4.58 (1.89) a	4.94 (1.58) a	4.91 (2.05) a	4.59 (2.13) a	4.35 (1.79) a	4.39 (1.73) a	4.22 (1.29) a	4.39 (1.70) a	5.09 (1.69) a	4.91 (2.12) a	4.46 (1.96) a
**Crumbly texture**	5.79 (2.00) a	5.39 (1.93) a	6.57 (2.40) a	6.11 (2.24) a	5.82 (2.57) a	5.45 (1.98) a	6.3 (1.94) a	5.33 (1.92) a	6.12 (1.82) a	5.57 (1.45) a	6.97 (2.20) a	6.87 (2.62) a	5.92 (2.36) a
**Oily mouthfeel ***	3.37 (2.22) a	3.25 (1.46) a	3.76 (2.58) a	4 (2.79) a	4.22 (2.32) a	4.35 (2.17) a	3.67 (2.38) a	4.22 (2.37) a	3.71 (2.05) a	3.62 (2.16) a	3.73 (2.75) a	4.19 (2.59) a	3.88 (2.34) a
**Aftertaste ***	3.43 (1.49) a	3.45 (2.03) a	4.12 (2.16) a	4.45 (2.44) a	3.65 (1.87) a	4.5 (1.97) a	3.14 (1.67) a	4.05 (1.97) a	3.59 (1.88) a	4.07 (2.39) a	3.8 (2.38) a	4.37 (2.73) a	3.58 (2.13) a

* Sweetness, bitterness, aftertaste, moist and oily mouthfeel were not statistically significant (*p* > 0.05), while other attributes had *p* < 0.05. ** FO = non-encapsulated fish oil and spry-dried emulsions formulated M = maltodextrin, W = WPC = whey protein concentrate, S = soy protein or P = pea protein as wall material, 1 = 1.5% oil, and 2 = 3.0% oil.

## References

[1] Turchini G.M., Nichols P.D., Barrow C., Sinclair A.J. (2012). Jumping on the omega-3 bandwagon: Distinguishing the role of long-chain and short-chain omega-3 fatty acids. Crit. Rev. Food Sci. Nutr..

[2] Saini R.K., Keum Y. (2018). Omega-3 and omega-6 polyunsaturated fatty acids: Dietary sources, metabolism, and significance—A review. Life Sci..

[3] Petsini F., Fragopoulou E., Antonopoulou S. (2018). Fish consumption and cardiovascular disease related biomarkers: A review of clinical trials. Crit. Rev. Food Sci. Nutr..

[4] Sioen I., Van Lieshout L., Eilander A., Fleith M., Lohner S., Szommer A., Petisca C., Eussen S., Forsyth S., Calder P.C. (2017). Systematic review on N–3 and N–6 polyunsaturated fatty acid intake in European countries in light of the current recommendations—Focus on specific population groups. Ann. Nutr. Metab..

[5] Christenson J.K., O’Kane G.M., Farmery A.K., McManus A. (2017). The barriers and drivers of seafood consumption in Australia: A narrative literature review. Int. J. Consum. Stud..

[6] Ismail A., Bannenberg G., Rice H.B., Schutt E., MacKay D. (2016). Oxidation in EPA- and DHA-rich oils: An overview. Lipid Technol..

[7] Regulation (EC) No 1924/2006 of the European Parliament and of the Council of 20 December 2006 on Nutrition and Health Claims Made on Foods. https://eur-lex.europa.eu/legal-content/en/ALL/?uri=CELEX%3A32006R1924.

[8] Serini S., Fasano E., Piccioni E., Cittadini A.R.M., Calviello G. (2011). Dietary *n*–3 polyunsaturated fatty acids and the paradox of their health benefits and potential harmful effects. Chem. Res. Toxicol..

[9] Vieira S.A., Zhang G., Decker E.A. (2017). Biological implications of lipid oxidation products. J. Am. Oil Chem. Soc..

[10] Ganesh V., Hettiarachchy N.S. (2016). A Review: Supplementation of foods with essential fatty acids-can it turn a breeze without further ado?. Crit. Rev. Food Sci. Nutr..

[11] Baldelli A., Wells S., Pratap-Singh A. (2021). Impact of product formulation on spray-dried microencapsulated zinc for food fortification. Food Bioprocess Technol..

[12] Encina C., Vergara C., Giménez B., Oyarzún-Ampuero F., Robert P. (2016). Conventional spray-drying and future trends for the microencapsulation of fish oil. Trends Food Sci. Technol..

[13] Damerau A., Ogrodowska D., Banaszczyk P., Dajnowiec F., Tańska M., Linderborg K.M. (2022). Baltic herring (*Clupea harengus membras*) oil encapsulated by spray-drying using a rice protein coating material—Effect of emulsion production parameters on emulsion and powder properties. J. Food Eng..

[14] Ogrodowska D., Laaksonen O., Tańska M., Konopka I., Linderborg K.M. (2020). Pumpkin oil addition and encapsulation process as methods to improve oxidative stability of fish oil. LWT Food Sci. Technol..

[15] Kaushik P., Dowling K., Barrow C.J., Adhikari B. (2015). Microencapsulation of omega-3 fatty acids: A review of microencapsulation and characterization methods. J. Funct. Foods.

[16] Jamshidi A., Cao H., Xiao J., Simal-Gandara J. (2020). Advantages of techniques to fortify food products with the benefits of fish oil. Food Res. Int..

[17] Jeyakumari A., Janarthanan G., Chouksey M.K., Venkateshwarlu G. (2016). Effect of fish oil encapsulates incorporation on the physico-chemical and sensory properties of cookies. J. Food Sci. Technol..

[18] Ahmed M., Akter M.S., Eun J.B. (2010). Impact of α-amylase and maltodextrin on physicochemical, functional and antioxidant capacity of spray-dried purple sweet potato flour. J. Sci. Food Agric..

[19] Wang Y., Liu W., Chen X.D., Selomulya C. (2016). Micro-encapsulation and stabilization of DHA containing fish oil in protein-based emulsion through mono-disperse droplet spray dryer. J. Food Eng..

[20] Chen Q., McGillivray D., Wen J., Zhong F., Quek S.Y. (2013). Co-encapsulation of fish oil with phytosterol esters and limonene by milk proteins. J. Food Eng..

[21] Di Giorgio L., Salgado P.R., Mauri A.N. (2019). Encapsulation of fish oil in soybean protein particles by emulsification and spray drying. Food Hydrocoll..

[22] Castejón N., Luna P., Señoráns F.J. (2021). Microencapsulation by spray drying of omega-3 lipids extracted from oilseeds and microalgae: Effect on polyunsaturated fatty acid composition. LWT Food Sci. Technol..

[23] Rodea-González D.A., Cruz-Olivares J., Román-Guerrero A., Rodríguez-Huezo M.E., Vernon-Carter E.J., Pérez-Alonso C. (2012). Spray-dried encapsulation of chia essential oil (*Salvia hispanica* L.) in whey protein concentrate-polysaccharide matrices. Food Eng..

[24] Gharsallaoui A., Saurel R., Chambin O., Voilley A. (2012). Pea (*Pisum sativum* L.) protein isolate stabilized emulsions: A novel system for microencapsulation of lipophilic ingredients by spray drying. Food Bioprocess Technol..

[25] Tamm F., Herbst S., Brodkorb A., Drusch S. (2016). Functional properties of pea protein hydrolysates in emulsions and spray-dried microcapsules. Food Hydrocoll..

[26] Damerau A., Ahonen E., Kortesniemi M., Puganen A., Tarvainen M., Linderborg K.M. (2020). Evaluation of the composition and oxidative status of omega-3 fatty acid supplements on the Finnish market using NMR and SPME-GC–MS in comparison with conventional methods. Food Chem..

[27] Hughes B.H., Muzzy H.M., Laliberte L.C., Grenier H.S., Perkins L.B., Skonberg D.I. (2012). Oxidative Stability and Consumer Acceptance of Fish Oil Fortified Nutrition Bars. J. Food Sci..

[28] Henna Lu F.S., Norziah M.H. (2011). Contribution of microencapsulated n–3 PUFA powder toward sensory and oxidative stability of bread. J. Food Process. Preserv..

[29] Meng Y., Cloutier S., Gaonkar A.G., Vasisht N., Khare A.R., Sobel R. (2014). Gelatin and other proteins for microencapsulation. Microencapsulation in the Food Industry—A Practical Implementation Guide.

[30] González A., Martínez M.L., León A.E., Ribotta P.D. (2018). Effects on bread and oil quality after functionalization with microencapsulated chia oil. J. Sci. Food Agric..

[31] Antoniewska A., Rutkowska J., Pineda M.M. (2019). Antioxidative, sensory and volatile profiles of cookies enriched with freeze-dried Japanese quince (*Chaenomeles japonica*) fruits. Food Chem..

[32] Starowicz M., Koutsidis G., Zieliński H. (2019). Determination of antioxidant capacity, phenolics and volatile Maillard reaction products in rye-buckwheat biscuits supplemented with 3β-d-rutinoside. Molecules.

[33] Demirkol A., Guneser O., Karagul Yuceer Y. (2016). Volatile compounds, chemical and sensory properties of butters sold in Çanakkale. J. Agric. Sci..

[34] Mallia S., Escher F., Schlichtherle-Cerny H. (2008). Aroma-active compounds of butter: A review. Eur. Food Res. Technol..

[35] Peterson D.G., Reineccius G.A. (2003). Characterization of the volatile compounds that constitute fresh sweet cream butter aroma. Flavour Fragr. J..

[36] Afoakwa E.O., Paterson A., Fowler M., Ryan A. (2009). Matrix effects on flavour volatiles release in dark chocolates varying in particle size distribution and fat content using GC–mass spectrometry and GC–olfactometry. Food Chem..

[37] Nightingale L.M., Cadwallader K.R., Engeseth N.J. (2012). Changes in dark chocolate volatiles during storage. J. Agric. Food Chem..

[38] Damerau A., Moisio T., Partanen R., Forssell P., Lampi A.-M., Piironen V. (2014). Interfacial protein engineering for spray-dried emulsions—Part II: Oxidative stability. Food Chem..

[39] Takeungwongtrakul S., Benjakul S., H.-kittikun A. (2015). Wall materials and the presence of antioxidants influence encapsulation efficiency and oxidative stability of micro-encapsulated shrimp oil. Eur. J. Lipid Sci. Technol..

[40] Christie W.W., Han X. (2010). Lipid Analysis. Isolation, Separation, Identification and Lipidomic Analysis.

[41] Damerau A., Kamlang-ek P., Moisio T., Lampi A.-M., Piironen V. (2014). Effect of SPME extraction conditions and humidity on the release of volatile lipid oxidation products from spray-dried emulsions. Food Chem..

[42] Tomic O., Luciano G., Nilsen A., Hyldig G., Lorensen K., Næs T. (2010). Analysing sensory panel performance in a proficiency test using the PanelCheck software. Eur. Food Res. Technol..

